# Clinical use of lenvatinib in patients with previous renal and/or hepatic impairment and radioiodine‐refractory differentiated thyroid cancer

**DOI:** 10.1002/cam4.5130

**Published:** 2022-10-06

**Authors:** Javier Martínez‐Trufero

**Affiliations:** ^1^ Medical Oncology Department University Hospital Miguel Servet Zaragoza Spain

**Keywords:** hepatotoxicity, lenvatinib, radioiodine refractory differentiated thyroid carcinoma, renal impairment

## Abstract

Lenvatinib is one the most active drugs in radioiodine‐refractory differentiated thyroid cancer (RR‐DTC) such that it has become an important therapeutic tool in tumor control and survival. Renal and hepatic impairments are common comorbidities in cancer patients. Treating these patients is a challenge that requires careful consideration. As a first approach to patients with RR‐DTC and renal or hepatic impairment, Summary of Product Characteristics recommendations for lenvatinib use and dose adjustments should be strictly followed. Close clinical and blood monitoring is the gold standard approach to optimizing lenvatinib's use during the whole course of treatment.

## 1 INTRODUCTION

Thyroid cancer is the eighth most frequently diagnosed cancer worldwide, and its incidence has increased during the past decades. Among all histologic subtypes, differentiated thyroid carcinomas (DTCs) that comprise mainly papillary, follicular, and poorly differentiated carcinomas represent around 90% of cases. Fortunately, the prognosis for DTC is very favorable in most cases, with almost 90% of patients having survival longer than 10 years when treated with surgery and radioactive iodine (RAI).^1^ Nonetheless, when DTC relapses and loses RAI avidity, the prognosis noticeably worsens. These patients, along with those diagnosed with other more aggressive subtypes such as anaplastic thyroid cancer (ATC) or medullary thyroid cancer (MTC), need different alternative treatments that nowadays rely mainly on tyrosine‐kinase inhibitors (TKIs).^2^

Lenvatinib mesylate (E7080) is an oral multitargeted TKI of vascular endothelial growth factor receptor 1–3, fibroblast growth factor receptor 1–4, platelet‐derived growth factor receptor α, RET, and KIT. Lenvatinib is indicated, among other tumors, for the treatment of recurrent or metastatic progressive, radioiodine‐refractory DTC (RR‐DTC). This approval relies on the results of a randomized phase 3 SELECT trial, where lenvatinib significantly improved median progression‐free survival (PFS) and overall response compared with placebo.^3^ Benefit of lenvatinib was seen in most patients, with a 64.8% objective response rate, and significant benefit in PFS compared with placebo (18.3 vs. 3.6 months).^3^ Although there are other drugs with proven efficacy in phase III studies versus placebo,^4^, ^5^ lenvatinib has a remarkable activity, achieving a long PFS, and thus becoming an important therapeutic tool that could play a key role in improving survival. Additionally, lenvatinib is being tested in other thyroid cancer subtypes such as ATC^6^ and MTC^7^ with promising results suggesting that it could be a potential therapeutic option for these subtypes. Unfortunately, a recent phase II trial with lenvatinib in ATC was stopped for futility as the minimum overal response rate (ORR) threshold of 15% was not met on the first interim analysis.^8^

## 2 HISTORICAL PERSPECTIVE

It is well known that cancer patients frequently have comorbid chronic conditions that share similar risk factors such as smoking, alcoholism, obesity, and sedentarism.^9^

Provided the long‐term disease control achieved with lenvatinib, patients need to maintain treatment for long periods of time. Therefore, control of toxicity becomes a crucial factor to optimize the clinical benefit of the drug. Although the toxicity profile of lenvatinib is manageable, its effects on kidney and liver function make its use in patients with renal and hepatic comorbidities a challenge, and the approach to its use can be decisive in being able to maintain its long‐term benefits in a considerable number of patients.

Regarding the pharmacokinetic properties of lenvatinib, it undergoes extensive hepatic and renal metabolism, either by enzymatic and non‐enzymatic pathways,^10^ with bile (64%) and urinary (25%) excretion.^11^ Therefore, although dose adjustment is not needed for patients with mild or moderate renal or hepatic failure, dose decrease is advisable for patients with severe renal or hepatic impairment.

Chronic kidney disease (CKD) and cancer are illnesses that are present at the same time in an increasing number of patients, being able to intefere with treatment compliance and efficacy.^12^ In the Renal Insufficiency and Anticancer Medications (IRMA) studies analyzing more than 5000 patients with cancer, it was estimated that approximately 50% of cancer patients could have a glomerular filtration rate (GFR) <90 ml/min/1.73 m^2^. In one of these studies it was shown that GFR <60 ml/min/1.73 m^2^ resulted in a decrease in survival from 25 to 16.4 months compared with patients with GFR ≥60 ml/min/1.73 m^2.^ ^13^ On the other hand, when CKD is present in a patient, it normally coexists with a median of five other long‐term comorbidities, leading to an increased risk of hospitalization of 2–3 times.^14^

In turn, in patients with chronic liver disease (CLD), liver‐related mortality is the leading cause of death, linked to a 1‐year mortality of 1%–3.4% in early stages of cirrhosis and 20–50% in advanced stages.^15^ Therefore, coexistence of cancer and CLD is a major therapeutic challenge, especially when chronic treatment with potentially hepatotoxic drugs is needed.

There are no specific prevalence data for liver dysfunction in cancer patients, but it is well known that hepatic impairment interferes with clearance of drugs eliminated either by hepatic metabolism or biliary excretion, and can also affect plasma protein binding.^16^ The effect of this on absorption of orally administered drugs such as TKIs can be the consequence of a reduced first‐pass effect, leading to increased bioavailability of these drugs in these patients.^17^

## 3 CURRENT SITUATION

According to the European Public Assessment Report (EPAR) recommendations for use of lenvatinib in RR‐DTC, no adjustment of initial dose based on hepatic function is required in patients with mild (Child‐Pugh A) or moderate (Child‐Pugh B) hepatic failure. In patients with severe (Child‐Pugh C) hepatic disease, the recommended initial dose is 14 mg once daily.^10^

Additionally, no adjustment of starting dose of lenvatinib based on renal function is required in patients with mild or moderate renal impairment. In patients with severe renal failure (GFR 15–29 ml/min/1.73 m^2^), the recommended starting dose is 14 mg once daily. Patients with end‐stage renal disease were not studied, so there are no evidence‐based data to establish recommendations in these patients. Further dose adjustments could be necessary based on individual tolerability for both renal and hepatic impairments.^10^

Among the adverse events of lenvatinib, some are critical to take into consideration when renal function is compromised. Related to renal function, in addition to renal impairment, toxicities directly linked to renal function described in DTC studies that should be closely monitored include proteinuria (reported in 33.7%), arterial hypertension (72.8%) and diarrhea (67.4%).^11^

Recent data from real‐world studies reported an incidence of 2% for liver dysfunction.^18^ In the EPAR recommendations, liver‐related adverse events of lenvatinib included hypoalbuminemia (9.6%), increased liver enzyme levels (alanine aminotransferase 7.7%, aspartate aminotransferase 6.9%), and serum bilirubin (1.9%).^19^ The median time to onset of liver dysfunction was 12.1 weeks. Grade 3 or higher liver toxicity occurred in 5.4% of lenvatinib‐treated patients. Liver‐related events were cause of dose interruptions and reductions in 4.6% of patients, and to permanent discontinuation in 0.4%.^10^ Interestingly, baseline hepatic impairment was associated with a higher rates of hypertension and palmar‐plantar erythrodysesthesia, and a higher incidence of Grade 3 or 4 hypertension, asthenia, fatigue, and hypocalcemia than was normal hepatic function.^10^

## 4 TREATMENT RECOMMENDATIONS

In order to be able to administer lenvatinib in RR‐DTC, provided that it is selected to achieve the best tumor control and survival, it is important to follow all the recommendations related to dose adjustments in the EPAR recommendations.^10^ In addition, it is useful to consider that dose adjustments based on pharmacokinetics do not always offer a high level of precision, since the physiological bases in patients with hepatic or renal impairment have not been completely elucidated, and the pharmacokinetics of a drug may display high inter‐individual variability. For this reason, therapeutic drug monitoring may be the gold standard approach, not only immediately after initiation of therapy, but also during the whole course of treatment.^10^ Also, other recommendations based on published studies and case experience can be helpful to prevent and manage toxicity whetreating a patient with RR‐DTC and comorbidities such as renal or hepatic impairment.

In relation to renal function, there are some useful recommendations to consider^20^:

- Other concomitant nephrotoxic therapies should be avoided if possible. Eventually, for a similar expected effectiveness, several drugs may be given, in which case the less toxic combination should be chosen.
- When a concomitant nephrotoxic drug is necessary, specific methods to avoid renal toxicity must be used, particularly aiming to prevent any dehydration.
- Special attention should be paid to the control of other toxicities such as diarrhea, proteinuria and hypertension that could affect renal function indirectly.
- Careful and continuous renal monitoring is crucial, both before and during treatment to identify early renal impairments, and to enable differential analysis of the cause of the renal event. Weekly clinical and blood monitoring is advisable, especially at the begining of treatment, with appropriate dosage adjustments when necessary.
- As there are no prospective data of patients with end‐stage renal disease undergoing hemodialysis, no evidence‐based recommendation can be offered, but there has been some experience with other TKIs in which treatment has been feasible and effective for most cases with close monitoring.^21^

Recommendations to consider for hepatic impairment^17^:

- As the liver metabolizes most drugs, hepatic impairment could decrease drug metabolism and alter exposure of a patient to drugs and their metabolites. For this reason, it is necessary to adjust doses of all concomitant drugs in addition to dose adjustments to the TKI, and to carefully monitor the patient in parallel with changes in hepatic function.^17^
- As for renal impairment, concomitant hepatotoxic drugs should be avoided whenever possible. When a hepatotoxic drug is required, hepatic function monitoring, adverse event surveillance, and careful dosage adjustment must be considered.^17^

## 5 CLINICAL CASE

**FIGURE 1:**
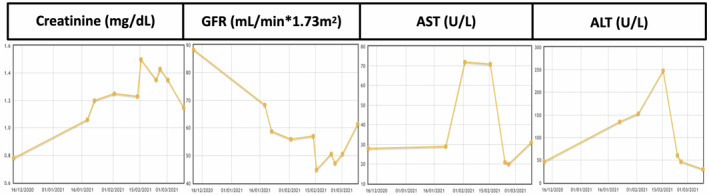
Kidney and liver enzymes showing worsening renal and hepatic function with later improvement. ALT, alanine aminotransferase; AST, aspartate aminotransferase; GFR, glomerular filtration rate.

The patient was a 74‐year‐old man with a medical history of paroxistic atrial fibrillation. His oncological history was as follows:

- October 2010: the patient underwent total thyroidectomy due to follicular carcinoma in the right thyroid lobe with 25% Hürthle cells.
- January 2011: he was treated with radioactive iodine (RAI; I131 100 mCi). Post‐therapy there were residual thyroid component and hyperuptake on cervical vertebra C3.
- September 2011: he was retreated with RAI (I131 200 mCi).
- August 2016: he had an episode of spinal cord compression at C3, which was treated with decompressive laminectomy with total function recovery. Pathological analysis revealed metastases of follicular carcinoma.
- December 2016: he was retreated with RAI (I131 150 mCi).
- June 2017: epidural progression of C3 metastasis was noted.
- July 2017: he underwent C3 surgical corporectomy and fixation.
- December 2017: local progression with incipient myelopathy was noted.
- December 2017: sorafenib 800 mg/day was initiated. After 3 days he presented with a grade III systemic dermatitis that prevented continuing with sorafenib.
- January 2018: external radiotherapy to C3 was done at a dose of 8 Gy.
- March 2018: lenvatinib 24 mg/day orally was initiated. The dose was reduced to 20 mg/day after 2 months because of hypertension. With this treatment, disease remained stable with good symptomatic control.
- January 2021: the patient was hospitalized with heart failure because of acute atrial fibrillation, which required electrical cardioversion. During this episode, due to concomitant medication, the patient developed both renal and hepatic impairment that necessitated cessation of lenvatinib for 14 days, after which, it was restarted at a dose of 14 mg/day. As shown in Figure 1, both renal and hepatic function worsened during that period, but later improved without having to stop lenvatinib.

This case demonstrates that close monitoring of patients with RR‐DTC is necessary and that doctors should be aware of incidental complications that could interfere with renal and hepatic function.

## 6 DISCUSSION

Lenvatinib is the most active drug in RR‐DTC, and has become a decisive therapeutic tool in tumor control and survival. Renal and hepatic impairment is a common comorbidity in cancer patients. In patients with renal or hepatic failure, following EPAR recommendations and close clinical and blood monitoring may be the gold standard approach to optimize lenvatinib use during the course of treatment.

As well as careful monitoring of toxic effects and symptoms, prior discussion with patients to provide them with the necessary information to promptly detect adverse events potentially associated with lenvatinib in the context of chronic liver or kidney disease is advisable in order to instate the appropriate support measures in line with patient preferences.

EPAR recommendations advise establishing upfront dose adjustments in case of severe renal impairment (GFR <60 ml/min/1.73 m^2^) or in severe hepatic impairment (Child‐Pugh C). However, provided that many patients sometimes gather in themselves several comorbidities, these adjustments can be considered even in less severe conditions on an individualized basis.

## 7 CONCLUSIONS

Liver and kidney comorbidities are therapeutic challenges when lenvatinib is going to be administered for advanced thyroid cancer. In order to optimize treatment and minimize dose interruptions, clinicians should closely follow the label recommendations and monitoring procedures. Careful and informed discussion with patients is highly recommended prior to starting therapy.

## FUNDING INFORMATION

The author has received an honorarium payment from Eisai Farmacéutica SA in line with ICMJE guidelines.

## CONFLICT OF INTEREST

Dr Javier Martínez Trufero has received honoraria and non‐financial support from Eisai, Lilly, Pharmamar, and Sanofi.

## Data Availability

Data sharing is not applicable to this article as no new data were created or analyzed in this study.
